# Genome-wide investigation of maize RAD51 binding affinity through phage display

**DOI:** 10.1186/s12864-022-08419-6

**Published:** 2022-03-12

**Authors:** Claire Milsted, Bo Dai, Nelson Garcia, Lu Yin, Yan He, Shahryar Kianian, Wojciech Pawlowski, Changbin Chen

**Affiliations:** 1 School of Life Sciences, Arizona State University, 427 E Tyler Mall, Tempe, AZ 85287 USA; 2 Department of Horticultural Science, University of Minnesota, 1970 Folwell Avenue, St. Paul, MN 55108 USA; 3 Calyxt Inc, 2800 Mount Ridge Rd, Roseville, MN 55113 USA; 4 School of Integrative Plant Science, Cornell University, 401 Bradfield Hall, Ithaca, NY 14853 USA; 5 National Maize Improvement Center of China, College of Agronomy and Biotechnology, China Agricultural University, Beijing, 100094 China; 6 Cereal Disease Lab, USDA-ARS, St. Paul, MN 55108 USA

**Keywords:** RAD51, Phage Display, Peptide, BRCA2, Transcription factor, Maize, *Zea mays*

## Abstract

**Background:**

RAD51 proteins, which are conserved in all eukaryotes, repair DNA double-strand breaks. This is critical to homologous chromosome pairing and recombination enabling successful reproduction. Work in Arabidopsis suggests that RAD51 also plays a role in plant defense; the Arabidopsis *rad51* mutant is more susceptible to *Pseudomonas syringae*. However, the defense functions of RAD51 and the proteins interacting with RAD51 have not been thoroughly investigated in maize. Uncovering ligands of RAD51 would help to understand meiotic recombination and possibly the role of RAD51 in defense. This study used phage display, a tool for discovery of protein-protein interactions, to search for proteins interacting with maize RAD51A1.

**Results:**

Maize RAD51A1 was screened against a random phage library. Eleven short peptide sequences were recovered from 15 phages which bound ZmRAD51A1 *in vitro*; three sequences were found in multiple successfully binding phages. Nine of these phage interactions were verified *in vitro* through ELISA and/or dot blotting.

BLAST searches did not reveal any maize proteins which contained the exact sequence of any of the selected phage peptides, although one of the selected phages had a strong alignment (E-value = 0.079) to a binding domain of maize BRCA2. Therefore, we designed 32 additional short peptides using amino acid sequences found in the predicted maize proteome. These peptides were not contained within phages. Of these synthesized peptides, 14 bound to ZmRAD51A1 in a dot blot experiment. These 14 sequences are found in known maize proteins including transcription factors putatively involved in defense.

**Conclusions:**

These results reveal several peptides which bind ZmRAD51A1 and support a potential role for ZmRAD51A1 in transcriptional regulation and plant defense. This study also demonstrates the applicability of phage display to basic science questions, such as the search for binding partners of a known protein, and raises the possibility of an iterated approach to test peptide sequences that closely but imperfectly align with the selected phages.

**Supplementary Information:**

The online version contains supplementary material available at 10.1186/s12864-022-08419-6.

## Background

The DNA double-strand break repair protein RAD51 is conserved in all eukaryotes but was initially identified in *Saccharomyces cerevisiae* [1]*.* It enables double-strand break repair, specifically catalyzing the alignment of the broken DNA ends to a double-stranded template, a process known as strand invasion. This homology-directed repair occasionally results in homologous recombination [1, 2].

In many eukaryotes including humans and Arabidopsis, RAD51-mediated DNA repair requires the DNA repair protein BRCA2. Mutations in the *BRCA2* gene are risk factors for breast and ovarian cancer in humans. BRCA2 proteins are large, containing several 39-amino-acid repeats, known as BRC repeats. The number and composition of these repeats varies between species. For example, both of the Arabidopsis BRCA2 proteins contain four BRC repeats while rice BRCA2 contains six BRC repeats [3–6]. Annotations in GenBank and pFAM suggest that the maize BRCA2 protein contains two BRC repeats ([7, 8] GenBank ID# AQK42450.1). In many species including humans and *Arabidopsis*, RAD51 binds to the BRC repeat region of the BRCA2 protein. The multiple BRC repeats allow multiple RAD51 molecules bind to a single BRCA2 molecule [3, 9, 10].

The crossing-over process in meiosis is a specialized form of homology-directed DSB repair [11]. This process in meiosis I allows DNA to be exchanged between chromosomes, leading to increased diversity and ensuring faithful chromosome pairing [12]. Therefore, mutations in *RAD51* genes cause meiotic and reproductive defects in many species [13–16]. Because maize is grown for its edible seeds, its reproductive processes, including meiosis, are of special importance. The function of RAD51 is less well characterized in maize than in Arabidopsis, and a single BRCA2 protein in maize has only been tentatively identified [8, 16]. Our understanding of the interactions of RAD51 proteins in maize is still at an early stage.

In maize, as in rice, there are five *RAD51* genes: *RAD51A1*, *RAD51A2*, *RAD51B*, *RAD51C*, and *RAD51D* [17, 18]. The two maize *RAD51A* genes, *RAD51A1* and *RAD51A2*, appear functionally redundant--a mutation in *RAD51A1* alone does not cause a macroscopically observable reproductive phenotype, but *rad51a1/rad51a2* double mutants are male sterile with very low female fertility [2]. The *RAD51A1/RAD51A2* split appears to be basal to all grasses and is present in both maize and rice [19]. In maize and Arabidopsis, RAD51 plays an additional role in pathogen resistance [20, 21]. The relationship between the defense function of RAD51 and DNA repair is unclear. There are numerous examples of plant cells having an increased rate of DSBs in response to pathogen infection since Mittler and Lam first identified this phenomenon in 1997 [22, 23]. Arabidopsis with loss-of-function mutations in either *RAD51* or *BRCA2A* had increased susceptibility to infection by *Pseudomonas syringae*, and when wild-type Arabidopsis plants were treated with the defense hormone INA, *AtRAD51* expression increased [20]. In Arabidopsis, RAD51 and RAD51D appear to play a direct role in inducing defense gene transcription and interact physically with transcription start sites. Additionally, RAD51D interacts physically with the defense gene suppressor SNI1 [20, 24–26]. SNI1 itself, although first discovered as a negative regulator of defense, is also involved in meiotic recombination; *sni1* mutant Arabidopsis displays increased rates of crossover formation [27]. Adding a maize *RAD51A1* transgene to Arabidopsis, rice, or tobacco increases resistance to selected pathogens [21].

Neither the overall binding affinity of maize RAD51A proteins nor the possible relationship between maize RAD51 proteins and transcription regulation has been investigated in the published literature. Therefore, this study aimed to identify proteins which may bind to RAD51A1. The phage display method, which casts a wide net for potential binding motifs, is well suited to this initial stage of investigation.

Phage display is a discovery-driven technique that screens for short peptides with high affinity for a given bait protein, in this case RAD51A1. When phage display is used in monoclonal antibody development, an antigen of interest is used as a bait protein while phages containing small fragments of the antibody protein on their surface are screened [28]. However, other experiments use a phage library composed of random amino acid sequences. Phage display harnesses the power of natural selection, since phages with phage coat additions that enable higher-affinity binding to the bait protein are more likely to be retained, grown in *E. coli*, and sequenced [29]. Phage display was chosen over a yeast-2-hybrid method for this study because of the possibility of using a large random library. Additionally, targeting specific short peptide sequences suggests future possible cytological experiments. Fluorescent-labeled peptides could be used as an alternative to fluorescent-labeled antibodies in protein detection [30–32].

## Results

### Properties of synthesized RAD51A1

**Fig. 1:**
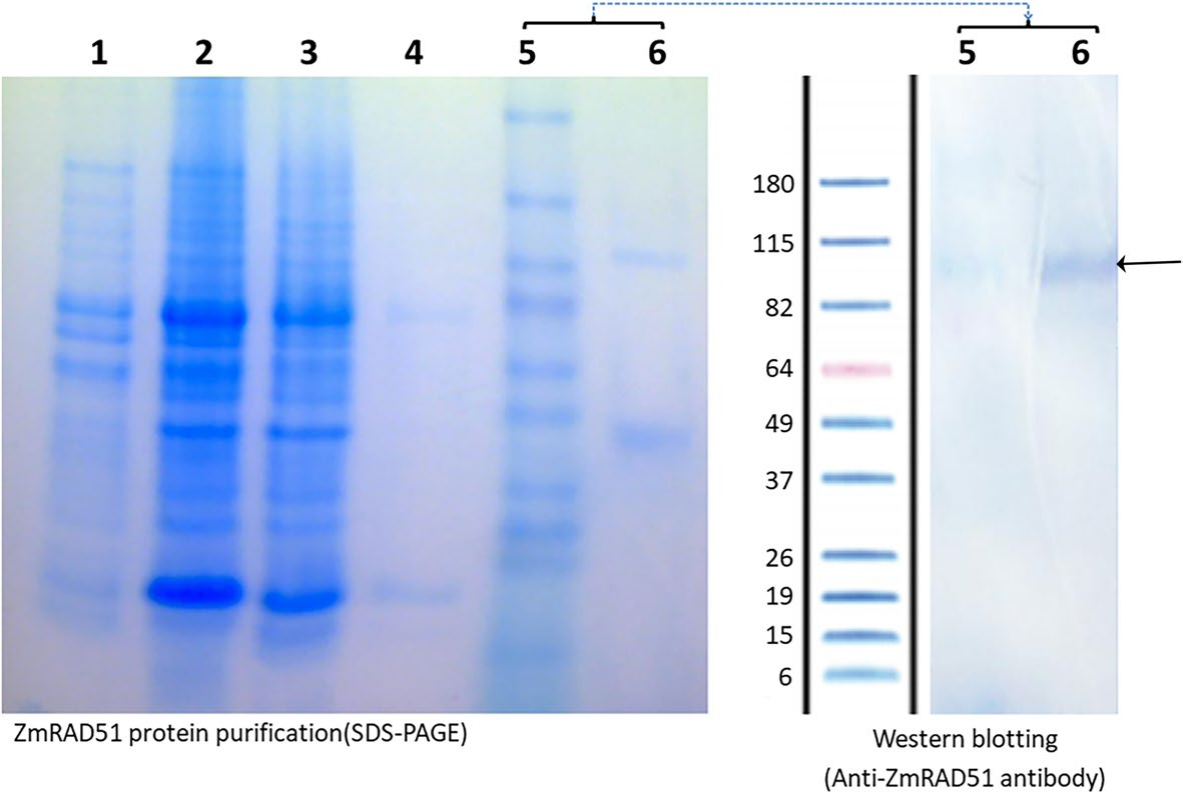
SDS-PAGE gel and western blot showing purification of RAD51A1. An SDS-PAGE gel is shown on the left, and a western blot probed with an α-ZmRAD51 antibody from rabbit is shown on the right. Gel lanes represent (1) supernatant, (2) pellet, (3) flow through, (4) wash, (5) marker, and (6) eluted protein. In the western blot on the right, lane (6) contains the eluted RAD51 protein probed with an α-ZmRAD51 antibody from rabbit. The RAD51A1 dimer band (92 KDa), indicated by an arrow, can be seen more clearly than the monomer band (46 KDa)

Two separate blots with different antibodies were used to verify the purity of the RAD51A1 grown in *E. coli*: one blot used an HRP-conjugated α-rabbit IgG secondary antibody from goat to bind to the α-ZmRAD51A antibody from rabbit; the other used an HRP-conjugated α-His-tag monoclonal antibody from mouse to bind to the His-tag of the synthesized RAD51A1. Both western blots revealed the presence of RAD51A1 dimers. The molecular weight of monomeric maize RAD51A1 is 46KDa, and there was a clear band of that size in the SDS-PAGE gel. However, in the western blot analyses, there was no signal at 46KDa but there was an apparent dimer band of about twice that size (Fig. 1). A separate blot contained also an apparent RAD51A1 trimer band. Furthermore, previous mass spectrometry analysis did confirm that the construct expressed full length anti-RAD51A1 before being used to generate anti-RAD51A1 antibody.

### Phage peptides selected by phage display

**Fig. 2:**
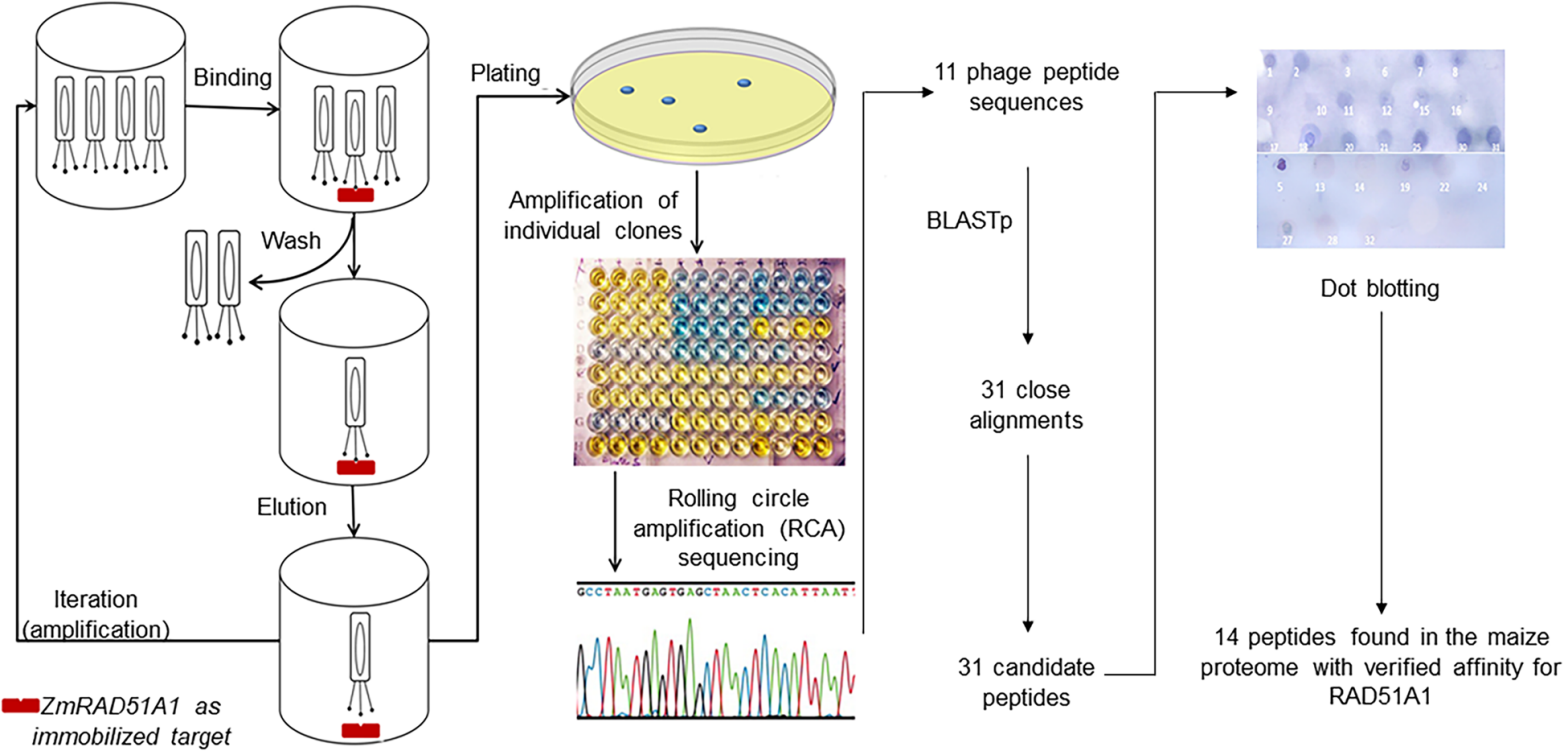
An outline of the methods used in the phage display and subsequent experiments. **A** A random phage display library was allowed to hybridize with RAD51A1, then washed to remove non-binding phages, and then eluted. Three rounds of selection were performed for a more rigorous screening of interactions. Following plating, phages selected for binding affinity for RAD51A1 were amplified and sequenced. **B** Maize peptides were selected for synthesis based on phage display results. These synthesized peptides were dot blotted for affinity with RAD51A1

Phages with coat proteins that enabled binding to maize RAD51A1 were grown in *E. coli* and the selected phages were sequenced (Fig. 2A). BLASTp and pFAM were used to analyze the phage sequences and search for either candidate maize proteins or conserved domains containing these amino acid sequences. When no such matches were found, 32 short (13–20 amino acids) peptides were designed from the primary sequences of maize proteins that imperfectly aligned with the selected peptides. For clarity, these peptides are referred to as “synthesized peptides” in contrast to the “phage peptides” selected in the phage display experiment. These synthesized peptides, whose sequences were found in the predicted maize proteome, were dot blotted to test their affinity for RAD51A1 (Fig. 2B).

The sequencing reaction only succeeded for 15 of the 24 single-plaque phages selected for affinity with RAD51A1; the remaining 9 reactions yielded low-quality results, possibly due to low concentrations of DNA. These 15 sequenced phages were found to contain 11 distinct peptide sequences, some of which were selected multiple times (Table 1). This included eight sequences of 16 amino acids. Sequencing also revealed three peptide sequences longer than the expected 16 amino acids: two 17-amino-acid peptides and one 18-amino-acid peptide. Analysis of these peptide sequences revealed three apparent recurring motifs: an isoleucine-tryptophan motif, a glutamate-tryptophan motif, and a more variable motif consisting of tryptophan in conjunction with glutamine, proline and/or serine. At least one of these motifs was present in each of the selected phage peptides. These motifs may be important to the ability to bind to maize RAD51A1. Tryptophan is present in all selected sequences except for phage peptide #24, suspected to be a false positive because it perfectly matches the sequence of a coat protein of the M13 bacteriophage used in the phage display experiment. The full list of peptides, along with ELISA and dot blot results, is shown (Table 1).

**Table 1:** Phage peptides identified after selection and amplification

Phage #	Peptide sequence	Phage clone(s)	ELISA affinity (native/denatured)	Dot blot
1	HLEYNAGYHSPATH**QWS**	1	−/−	–
2	TWHDTFHAKGTG**QSWS**	5, 6	+/+	+
3	LSIS**IW**FFPXESSHKS	8	−/−	+
4	HQTPMH**IW**PEHKLGHR	9	−/+	–
5	SSLGQP**WI**GAPRAYPW	10	−/+	–
6	HY**SQS**LTYTWPKFGEI	13	−/+	+
7	TSNTTP**WQ**TS**WE**LMYA	14, 15, 21	+/+	+
8	HHTHWHT**SQ**D**WE**PQPHAS	16	+/+	–
9	YTGLHYQPWWPDVVQG	20	+/+	+
10	D**EW**NEMGNIP**SQL**IMA	22, 23	+/+	–
11	AAEGDDPAKAAFN**SLQ**A	24	−/−	–

Phages containing these peptides showed the ability to bind to maize RAD51A1 *in vitro*. Some sequences were selected multiple times. Recurring motifs are underlined and bolded. ELISA affinity and Dot blot refer to the results of experiments to verify the affinity of the phages for RAD51A1. ELISA affinity refers to affinity for either native or denatured RAD51A1. “+/+” signifies that the phage had affinity for native and denatured RAD51A1; “−/+” signifies affinity only for denatured RAD51A1 and so on. Dot blot result refers to binding affinity for native RAD51A1 as determined by dot blot. ELISA and dot blots verified RAD51A1 affinity of most selected phages

Binding affinity for RAD51A1 was verified for all the selected phages whose additional peptide sequences had been sequenced at 16 amino acids in length; only phages 1, 16, and 24 did not demonstrate *in vitro* affinity with RAD51A1 (Table 1). In the ELISA, phages’ affinity for native and denatured RAD51 was compared to their affinity for a BSA negative control. Wells with 1.5-fold or greater increase in optical density above control were coded as positive. Phages 4, 5, 6, 7, 14, 15, 16, 20, 21, 22, and 23 were positive against both native and denatured RAD51A1 protein; four additional phage strains [9, 10, 13, 17] were positive against denatured protein only (Fig. S1). Peptide 3 did not clear the 1.5-fold threshold in the ELISA, possibly due to high affinity for the BSA control, but demonstrated affinity in the dot blot and in the western blot (Figs. S1, S2). Each phage/protein combination was repeated two or three times, and phages found to contain the same peptide sequences as other phages were still tested separately.

Phages were also incubated in dot blots to further confirm affinity for RAD51A1. To investigate possible affinity with other proteins produced alongside RAD51A1, phages were incubated with the supernatant, pellet, flow through, and two subsequent elutions of native RAD51A1. Phages 5, 8, 13, 14 and 20 bound to the protein supernatant, protein in pellet and flow through and elution 1. This unexpected binding to earlier steps in the extraction of the RAD51A1 protein may be due to presence of RAD51A1 in these stages (see the possible RAD51A1 dimer bands in the supernatant, pellet, and flow-through in Fig. 1), or since these are M13 bacteriophages, liable to also interact with some proteins from their natural host, *E. coli*. The second eluted fraction did not bind *in vitro* to any phages, possibly due to a high concentration of the base imidazole in the elution buffer raising the pH and causing the protein to fail to immobilize (Fig. S2).

### Bioinformatic analysis of phage peptide sequences

**Fig. 3:**
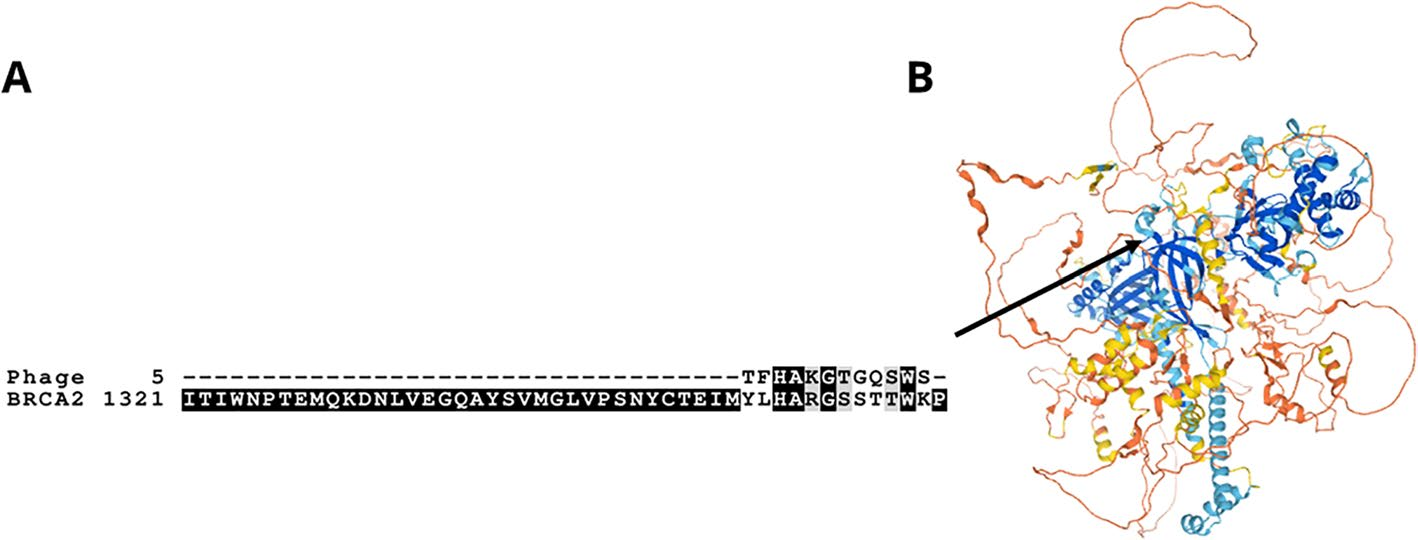
Maize BRCA2 residues 1358–1367 align with a portion of phage peptide #2. Peptide #2 was selected twice in the phage display experiment. The aligning region of maize BRCA2 is annotated as RPA_2b-aaRSs_OBF_like Superfamily in GenBank. **A** The amino acid sequence at the site of alignment. Black represents an alignment between two identical amino acids and grey represents a positive alignment. The E-value when this specific alignment is BLASTed is 0.5, with 44% identity. Amino acids aligned using ClustalW. Diagram produced using Boxshade (http://www.ch.embnet.org/software/BOX_form.html) **B** Location within the structure of maize BRCA2, as predicted by Alphafold [33]. See Uniprot s# A0A1D6J355. Dark blue represents very high confidence (pLDDT >90) and orange represents very low confidence (pLDDT <50). This region’s structure is predicted at “confident” to “very high” confidence

BLASTp searches for the peptides selected in the phage display did not reveal any known or predicted maize proteins aligning perfectly to the full peptide sequence of any of the selected peptides, but several alignments to maize proteins, with E-values between 0.26 and 8.9, were identified (Table 2). Several of the closest alignments were to DNA-associated proteins such as transcription factors, proteins related to defense, and uncharacterized proteins. Additionally, a MEGA7 muscle alignment and a BLAST pairwise alignment to the BRCA2 revealed that a portion of phage peptide #2, selected twice, aligns imperfectly to a region of the BRCA2 protein with 44% identity and an E- value = 0.5 when aligned with the full-length Zm00001d024953_P024 sequence, or an E-value of 0.079 when aligned to the RPA_2b-aaRSs_OBF_like domain specifically, see Fig. 3.

**Table 2:** Maize proteins aligning to the selected phage peptide sequences shown in Table 1

Phage peptide #	Aligned protein annotation	E-value	Query coverage	Percent identity	NCBI accession
1	Uncharacterized protein LOC100381696 isoform X1	2.4	47%	87.5%	XP_008673086.1
1	Integrator complex subunit 9	2.4	47%	87.5%	PWZ34425.1
1	Mitogen-activated protein kinase kinase kinase A	4.9	47%	87.5%	PWZ31635.1
4	Boron transporter 4	2.9	43%	85.71%	ONM62448.1
7	Putative ribosomal protein S4 (RPS4A) family protein	1.4	43%	85.71%	AQK78541.1
7	Disease resistance gene analog PIC21	4.1	50%	75%	AAC83569.1
7	Disease resistance protein RPS2	4.2	50%	75%	PWZ33587.1
7	Uncharacterized protein LOC109944554	5.9	43%	88%	XP_020404914.1
7	Glucuronoxylan 4-O-methyltransferase 2	5.9	62%	90%	ONL96892.1
7	Pectate lyase 12	5.9	50%	87.5%	ONM06095.1
8	Heat stress transcription factor B-4	8	88%	50%	PWZ13703.1
9	Phosphoinositide phosphatase SAC6	2.1	56%	71.43%	AQK71706.1
9	Prolyl endopeptidase isoform X2	4.2	43%	85.71%	XP_008669028.1
9	Prolyl oligopeptidase family protein	4.2	43%	85.71%	ONM15068.1
10	Citrate synthase 1	0.26	62%	70%	AQK69670.1
10	Transcription regulator	4.2	50%	87.5%	ONM03717.1
10	CCR4-NOT transcription complex subunit 1	4.2	75%	87.5%	PWZ58289.1
10	Uncharacterized protein LOC100502409 isoform X10	4.2	75%	87.5%	XP_020399402.1
10	Uncharacterized LOC100383837	4.2	75%	87.5%	NP_001349390.1

A cutoff E-value of 8 was used. Hypothetical proteins not supported by EST or cDNA, suspected false positives (phage peptide #11), and cases where multiple proteins with similar or identical annotation were listed are not included. This table is based on maize genome sequence draft v5

BLASTing the selected phage peptides against all non-redundant proteins from all species in the NCBI database resulted in only one perfect match, between the 17-mer peptide #11 from phage clone #24 and the coat protein of the *E. coli* bacteriophage virus M13 (Table S1). This perfect match to the bacteriophage used in the phage display experiment suggests that phage peptide #11 is a false positive selected due to off-target amplification of viral DNA. Additionally, this phage did not bind to the RAD51A1 protein in either ELISA or dot blot assays (Table 1). Several of the selected sequences revealed weak alignments (E-values of 1.6–8.9) with DNA-associated proteins in other organisms. No putative conserved domains were detected by either BLAST or pFAM analysis; this may be because these tools were not designed for such short input sequences.

### Synthesis and binding affinity of selected maize peptides

An initial search, performed in 2015 using maize genome version 3, revealed several imperfect alignments between selected peptides and the maize proteome. These close alignments led to the selection of 32 peptides to be synthesized by GenScript (genscript.com; GenScript USA Inc., Piscataway, NJ 08854, USA) (Table 3). Dot blots showed that 14 of these peptides had *in vitro* affinity for RAD51A1 (Fig. S3). A list of the maize proteins which contains these positive synthesized peptide sequences can be found in Table 4.

**Table 3:** Synthesized peptides used in the final dot-blotting experiments

	Synthesized peptide sequence	Derived from phage peptide #	Dot blot
**1**	**EALYHSPNVATHS**	**1**	**+**
**2**	**RGGYNGGYRGPAA**	**1**	**+**
**3**	**RTWLASYHLRGTAQTW**	**2**	**+**
4	KISWNETFHSVVEA	2	–
5	HSLTFTWHDAFKG	2	–
6	SMISVFFPNESVQKS	3	–
**7**	**AGARAWRWPEHELGA**	**3**	**+**
**8**	**SKLGHRLSVETGPKPSRGK**	**3**	**+**
9	RPMRKWSESKLGTEL	4	–
10	GHRSEETHQEGATER	4	–
**11**	**RIGEPWIGVPSSG**	**5**	**+**
**12**	**RPGPSPRLGQPWRLG**	**5**	**+**
13	GTPWISAPKNTLR	5	–
14	ANIYSRGVTYEWHKF	6	–
**15**	**QTLMYTATWPKEVRK**	**6**	**+**
16	PWEPSWELFREKVGD	7	–
17	DRGWSIRAWELMY	7	–
**18**	**EHVWQTSWAISTRFVG**	**7**	**+**
**19**	**SHTSTSNATTPRSFD**	**8**	**+**
**20**	**KGGGVPQPHLSAEAR**	**8**	**+**
21	EPQPHSNGTASPAPPA	8	–
22	ANHTSQDEPHGGAL	8	–
23	SAAPPQPHANAMNL	8	–
24	EQFVVIGSAETVPPRHSL	8	–
**25**	**RPRWPDVQGRSYASR**	**9**	**+**
**26**	**GGAEMGLQTQLLMANA**	**10**	**–**
**27**	**DDWTNMAMVGISPQILMKSA**	**10**	**–**
**28**	**AAEEDAAAPARAASLQA**	**11**	**–**
29	TFAFNSLQVSFSFE	11	–
**30**	**GDQEDSAAAFPSLQA**	**11**	**+**
**31**	**SKDDPAKEAASFTS**	**11**	**+**
32	SSLGQPWRGALLLPAPRA	11	–

These 32 peptides were derived from close alignments between the selected phage peptides shown in Table 1 and known or predicted proteins found in the maize proteome. Dot blot refers to binding affinity for native RAD51A1. Interactions demonstrating affinity are bolded. More information on the peptides found to have affinity with RAD51A1 in the dot blot assay can be found in Table 4

**Table 4:** Maize proteins containing the synthesized peptides which bound RAD51A1 in vitro

Synthesized peptide #	Derived from phage peptide #	Matching protein(s)
**1**	**1**	**Probable WRKY transcription factor 63XM_008671716**
2	1	Plasminogen activator inhibitor RNA-binding protein, NM_001371832
**11**	**5**	**Two heat stress transcription factors: XP_008670235, heat stress transcription factor B-1, and ACG38635, heat shock factor protein 4**
15	6	Three DEAD-like helicases: putative DEAD-box ATP-dependent RNA helicase family protein XP_020405421, putative DEAD-box ATP-dependent RNA helicase family protein AQK96024, and DEAD-box ATP-dependent RNA helicase 40-like NP_001348908
18	7	Prolyl-tRNA synthetase, NP_001149927
19	8	LRP1 - lateral root primordia 1, XP_008656860
**20**	**8**	**DUF455 family protein, ONM28467**
25	9	Suspected annotation error
**30**	**11**	**Two bHLH transcription factors: transcription factor MYC2 XP_020402137 and uncharacterized protein NP_001168647**
31	11	Ten Elongation factor 1-alpha-like proteins: AQK85106, NP_001152668, XP_008656156, XP_008656153, NP_001338678, NP_001105587, NP_001105933, XP_035815740, AQK85098, Q41803

These peptides were designed based on regions of known or predicted maize proteins with close alignment to selected phage peptides from Table 1. Of these 32 synthesized peptides, 14 successfully bound RAD51A1. Putative transcription factors are bolded. Annotations are based on maize B73 reference genome v5. Synthesized peptides 3, 7, 8, and 12 bound RAD51A1 but were derived from putative peptide sequences now thought to be pseudogenes based on more recent annotations; these are not shown

## Discussion

### RAD51A1 dimerization may influence peptide binding affinity

RAD51 proteins are known to polymerize in many species including *Saccharomyces cerevisiae* and the archaeon *Pyrococcus furiosus* [9, 34]. Previous Western blot analysis has shown a signal from the maize RAD51A1 dimer using anti-HsRAD51 antibody [35]. Both western blots revealed a RAD51A1 dimer band at 92 KDa with a stronger signal than the expected monomer band at 46 KDa. This stronger signal could be due to the higher rate of formation of dimers, increased solubility of dimers, or increased affinity of the antibodies for the dimer form— although the dimer signal was stronger regardless of whether the α-ZmRAD51 antibody or an α-His-tag antibody was used, suggesting that the specific antibody is not the cause of the bias towards the dimer signal. This result is in line with previous studies using anti-human RAD51 antibody to detect maize RAD51A1, which were successful but showed multiple bands, indicating RAD51 dimerization [35]. The construct pET28b to express the full length ZmRAD51A1 used in this study was built to generate the anti-ZmRAD51A1 antibody. The product of pET28b was confirmed using mass spectrometry analysis as the full length of ZmRAD51A1 protein.

### Properties of peptides found to confer phage affinity for maize RAD51A1

Of the 11 distinct phage peptides, three (#2, #7, and #10) were selected multiple times. Given the number of single-plaque phages sequenced (24) and the size of the phage library (2.6E10 peptides), repeated selection of a given peptide would be unlikely due to chance. Furthermore, the three phages that were selected multiple times all displayed affinity for both native and denatured RAD51A1 in ELISA assays; phages #2 and #7 also bound to native RAD51A1 in dot blots. Phage peptide #2, selected twice, aligns to a binding region of maize BRCA2 (Fig. 3). This alignment between a binding domain of a putative RAD51A1-interacting protein (BRCA2) and a phage peptide selected twice for affinity with RAD51A1 may help elucidate the *in vivo* interaction between maize RAD51A1 and BRCA2.

The presence of both DNA-associated and plant defense related proteins in both the maize-specific BLASTp hits (Table 2) and the general BLASTp hits (Table S1) supports the idea of a dual function for RAD51 in DNA repair and plant defense. However, the E-values of these alignments (0.26–8.9) indicate that alignments of this quality might be expected by chance.

Some recurring short motifs were initially noted among the peptides generated by phage display (Table 1), but this may have been due to chance. In a later experiment, the majority of the synthesized peptides which bound RAD51A1 did not contain these short motifs. Similarly, the initial observation that tryptophan was present in every selected phage peptide (with the exception of the suspected false positive, phage peptide #11) did not hold true in the synthesized peptides, many of which bound RAD51A1 in a dot blot despite lacking tryptophan. However, despite this difficulty uncovering recurring motifs, the phage display results did identify interactions for further investigation, including those selected for the synthesized peptide experiment.

### Maize peptides show affinity for RAD51A1

Since there were no perfect matches between the phage peptides and the primary sequence of any known maize proteins, this study included an additional *in vitro* experiment with non-phage synthesized peptides. Peptide sequences were selected from maize proteins that aligned with, but did not perfectly match, the phage peptides (Tables 3 and 4). Selection of peptides for synthesis occurred before the release of the fourth and fifth versions of the maize genome, and the results were slightly different from what was found in later analyses; the fact that these annotations were later removed suggests that some of the proteins initially identified may have been pseudogenes (Table 3).

Sequences from two transcription factors possibly involved in defense bound to RAD51A1 in dot blot experiments. Synthesized peptides 1 and 2 successfully bound to RAD51A1 *in vitro* (Tables 3 and 4), unlike phage peptide #1 (Table 1), from which they were derived. Synthesized peptide #1 was designed to match part of the amino acid sequence of a WRKY transcription factor. These transcription factors (aligning with peptides 1 and 2), which contain a conserved amino acid motif beginning with WRKY, can be involved in regulating response to a range of biotic and abiotic stresses in plants, including pathogens [36]. The negative recombination regulator SNI1, which also serves as a defense gene regulator, appears to interact physically with several WRKY transcription factors in Arabidopsis [25, 27, 36, 37]. Synthesized peptide #30, which was developed from the transcription factor MYC2, also binds RAD51A1 *in vitro*. In Arabidopsis, MYC2 serves as a negative regulator of plant defense in the salicylic acid pathway and a positive regulator of the jasmonic acid pathway [38]. In both proteins (transcription factors WRKY and MYC2), the region containing the selected peptide is not a known conserved domain, nucleotide-binding or otherwise.

Sequences from two other proteins thought to be involved in transcription regulation bound to RAD51A1 in dot blots. Synthesized peptide #20 was derived from ONM28467, a Domain of Unknown Function (DUF) 455 protein with a Bromodomain extra-terminal (BET) domain. BET domains are associated with transcription factors which may also play a role in remodeling acetylated chromatin to enable transcription [39, 40]. Previous annotations noted similarity between this protein and the Arabidopsis transcription factor GTE4. While this locus is not the most similar maize gene to Arabidopsis *GTE4*, a BLAST search of the Arabidopsis proteome revealed that it does align closely to an Arabidopsis GTE4 isoform (E-value = 5e-80, 58% identities, 68% positives). In Arabidopsis, GTE4 is involved in controlling the mitotic cell cycle; *gte4* mutants have many aneuploid somatic cells due to endoreduplication taking the place of mitosis [41]. In mice BET domain containing proteins are required for both mitosis and spermatogenesis [40]. Synthesized peptide #11 is derived from a sequence in a protein annotated as Heat Stress Transcription Factor B-1 and also matches the sequence of another transcription factor annotated as Heat Shock Factor Protein 4.

### Strengths and weaknesses of the phage display method

This investigation into specific sequences that enable affinity for RAD51A1 involved short peptide sequences as opposed to full-length proteins which fold themselves into higher-order structures. Therefore, the short peptide sequences selected in the phage display may not match the primary amino acid sequence of the proteins that interact *in vivo* with the protein of interest. BLAST and pFAM algorithms were not designed for such short input sequences, and there is a potential for false positive BLASTp hits due to chance. The second experiment, investigating the binding affinity of short, synthesized peptides which perfectly matched portions of the primary sequences of maize proteins (Tables 3 and 4), was intended to validate some of these possible interactions.

The strength of phage display compared to other methods—the ability to screen a large factorial library of 2.6E10 sequences—is also a weakness since this increases the likelihood of selecting proteins not produced in maize. Other methods, such as a competitive yeast-2-hybrid assay with a specific cDNA library, avoid this problem but do not screen as many possible prey sequences.

Finally, these peptides could be used as alternatives to antibodies to verify the presence of RAD51 *in vitro* or even to localize it *in vivo*. Discovery of the 14 synthesized peptides with binding affinity to RAD51A1 provides a great opportunity for further screening of peptides that can be synthesized and conjugated with fluorescent markers. These modified peptides could be used to directly localize RAD51A1 without an α-ZmRAD51 antibody. Various systems using small fluorescent-labeled peptides as an alternative to antibodies have shown promising results *in vitro* and *in vivo* [30–32]. Antibody development is costly, and immunolocalization often relies on secondary antibodies, which limit the combinations of primary antibodies that can be co-localized together. Furthermore, the cell wall can interfere with large molecules such as antibodies entering the cell. Short fluorescent-labeled peptides could therefore provide an alternative to antibodies for immunolocalization.

## Conclusion

The phage display results together with the experiments with synthesized peptides show that the maize RAD51A1 protein has the capacity to bind to a variety of different small peptides. The alignment of phage peptide #2 with a putative binding domain of BRCA2 suggests that this may be the region of BRCA2 to which RAD51A1 binds. DNA-binding and plant defense related proteins were found among the set of proteins closely aligning with the phage peptides and the set of proteins containing the synthesized peptides; this supports the idea of a dual function for RAD51 in DNA repair and plant defense.

The most intriguing finding of this series of experiments was that a synthesized peptide sequence found in the defense-related transcription factor MYC2, as well as a second bHLH transcription factor of uncharacterized function, binds to RAD51A1 in a dot blot experiment. A synthesized peptide sequence found in a WRKY transcription factor, from a family of transcription factors often involved in defense, also bound to RAD51A1. This is congruous with the fact that in Arabidopsis, RAD51 appears to function as a transcription factor during defense; interactions with defense-related transcription factors would help to explain this function. The synthesized peptides have an apparent pattern of aligning to regions of transcription factors that are not conserved domains; RAD51A1 may bind to the non-conserved portions of these proteins, leaving their conserved domains free to bind other ligands including DNA. In contrast, phage peptide #2 aligns with a conserved binding domain of maize BRCA2.

At present, the most common uses of phage display are in medicine and immunology, including developing antibodies [29, 42, 43]. However, phage display is also a powerful tool for basic science research in other organisms, including plants [44]. Phage display can help to discover novel protein-protein interactions *in vitro*, as well as the motifs or domains that may drive known interactions. For example, these phage display findings suggest a possible RAD51 binding site in a region of maize BRCA2 that aligns closely with a phage peptide sequence that was selected twice for affinity with RAD51. The ability to screen a large random library is especially promising for understanding and predicting the binding affinities of highly polymorphic proteins such as defense genes that are in flux due to an evolutionary arms race.

## Methods

### Key reagents

The *E. coli* strain BL21(DE3) was purchased from Novagen (Gibbstown, NJ). The plasmid pET28b carrying the maize *RAD51A1* gene with the addition of a C-terminal His-tag, the RAD51A1 protein purified from *E. coli* and denatured and stored in a mixture of SDS and β-mercaptoethanol. The product of pET28b was previously verified to be RAD51A1 using mass spectrometry. The α -ZmRAD51 antibody from rabbit and the a-rabbit secondary antibody were provided by Dr. Wojciech Pawlowski at Cornell University. The α -ZmRAD51 antibody was generated using the full-length maize RAD51A1 protein from the pET28b plasmid and has been successfully used in both maize RAD51A1 immunolocalization and ZmRAD51A1-ChIP sequencing [18, 45]. Ni-nitrilotriacetic acid (Ni-NTA) agarose was purchased from Qiagen.

The horseradish peroxidase (HRP) conjugated α-His-tag monoclonal antibody from mouse and the Ultra TMB-Blotting Solution were purchased from Thermo Fisher (Waltham, MA). The HRP-conjugated α-rabbit antibody from goat was purchased from Abcam.

A library of M13 bacteriophages expressing random additional 16-amino acid surface peptides was purchased from Creative Biolabs (Piscataway NJ). *E. coli* strain ER 2738 was obtained from New England Biolabs (Ipswich, MA). The LB medium, isopropyl-β-D-thiogalactoside (IPTG), 5-bromo-4-chloro-3-indolyl-β-D-galactoside (X-gal), polyethylene glycol 8000, tris-HCl, and tetracycline were obtained from Sigma-Aldrich (St. Louis, MO). Synthesized peptides were synthesized by Genscript (Piscataway, NJ, USA).

### Expression and purification of His-tagged RAD51A1

The plasmid pET28b carrying the RAD51A1 gene containing a C-terminal His-tag was transformed into BL21(DE3) *E. coli*. The BL21(DE3) cells were grown at 25 °C in 2YT medium supplemented with 50 μg/mL kanamycin to select for the pET28b plasmid. The presence of the RAD51A1 sequence in the BL21(DE3) cells carrying pET28b was validated by sequencing the ORF of the plasmid. When the OD_600_ of the culture reached a value of 0.6, IPTG was added to a final concentration of 1 mM. The induced cell culture was grown for three days at 25 °C. The cell cultures were then incubated on ice for 15 min and harvested by centrifugation (4 °C; 30 min; 4000 rpm). The cell pellets were frozen at −20 °C.

The RAD51A1 protein was extracted from the *E. coli* pellet. The cell pellets were thawed at 4 °C and re-suspended in lysis buffer A (50 mM NaH_2_PO_4_, 300 mM NaCl, 10 mM imidazole, 10% glycerol, pH 8.0) with 1 mg/l lysozyme. The cells were then disrupted by sonication in lysis buffer A. Sonication consisted of 10 short bursts of 10 s, each followed by a 30-s cooling interval. The cell lysate was centrifuged (4 °C; 90 min; 4300 × *g*), and the supernatant was applied to a Ni-NTA agarose column (Qiagen.com, Catalog #30210) equilibrated with lysis buffer A. After the column was washed with 25 column volumes of buffer B (20 mM imidazole, 50 mM NaH_2_PO_4_, 300 mM NaCl, pH 8.0), the RAD51A1 protein was eluted with buffer C (300 mM imidazole, 50 mM NaH_2_PO_4_, 300 mM NaCl, pH 8.0). This yielded six 1.5 mL aliquots of eluted RAD51A1. Purity of the eluted RAD51A1 was verified by Western Blot. The remaining eluted RAD51A1 was stored at −20 °C with 20% glycerol.

### Western blotting

The eluted proteins were separated by 4–12% SDS-PAGE. Samples of native RAD51A1 from *E. coli*, eluted in buffer C were loaded in one 12-well NuPAGE gel (Life Technologies). BenchMark™ Pre-stained Protein Ladder (Life Technologies) was used as marker. Proteins were transferred onto a nitrocellulose membrane using the iBlot® 2 Gel Transfer Device. The resulting blots were blocked with 5% BSA in PBS. One blot was probed with a 1:1000 α-ZmRAD51 antibody for one hour, and subsequently incubated with an HRP-conjugated α-rabbit IgG secondary antibody for one hour. A second western blot was performed using an HRP-conjugated α-HisTag monoclonal antibody. The membranes were washed six times with phosphate-buffered saline with Tween® detergent (PBST). Each PBST wash consisted of a five-minute incubation at room temperature on a shaker at 150 rpm. The Ultra TMB blotting substrate was used for color development.

### M13 phage display

The library of 2.6E10 M13 bacteriophages were tested for affinity with RAD51A1. This experiment used RAD51A1 produced in *E. coli*. Three rounds of bio-panning were performed for the selection of RAD51A1-binding peptides (Fig. 2, Fig. S2).

For the first round of selection, 10 μg/mL RAD51A1 protein, previously denatured in SDS and β -mercaptoethanol and suspended in a pH 9.4 coating buffer, was added to each well of a 96-well ELISA plate and incubated overnight at 4 °C. A blocking solution of 3% BSA in PBST, and plates were incubated at room temperature for two hours to prevent non-specific binding. 2E-12 mol of the phage particle solution were added to each well.

The plate was incubated for one hour at room temperature in 2% BSA. Plates were washed three times with PBST for five minutes on a shaker at 150 rpm. Phages were eluted by a 10-min incubation at room temperature with 10 mM acetic acid (pH 2.2) and pH-neutralized with 1 M Tris-HCl (pH 8.0).

For the second round of selection, the eluted phages from the first round of selection were screened for affinity a second time in a similar 96-well plate trial. This was similar to the first round of selection but with the concentration of denatured RAD51A1 was decreased to 1 μg/mL. The plates were washed six times with PBST as described above.

For the final round of selection, the eluted phages from the second round of selection were screened for affinity with 1 μg/mL denatured RAD51A1 immobilized on a small PVDF membrane disc to prevent selection of phages with affinity for the plastic material of the ELISA plate. The membrane was placed in a flask and incubated for one hour in 2% BSA and washed three times with PBST for five minutes on a shaker at 150 rpm. Phages were eluted in acetic acid and Tris-HCl as in the previous rounds of selection.

### Phage amplification, purification, and titration

*E. coli* strain ER2738 cells were cultured in LB broth medium with 20 μg/mL of tetracycline. The cells were grown until the early-log phase (OD_600_ = 0.5), then used as host cells. The phages from the phage display, suspended in acetic acid and Tris-HCl, were added to the culture and co-cultured in a shaking incubator for four hours at 37 °C at 200 rpm.

The culture was centrifuged at 8000 rpm for 10 min to remove *E. coli* ER2738 cells. The supernatant solution was added to a PEG/NaCl solution at a 5:1 ratio and incubated at 4 °C for two hours. The resulting phage precipitate was recovered by centrifuging the sample at 4000 rpm for 30 min, removing the supernatant. The pellet was re-suspended in 2 mL of Tris-buffered saline (pH 8.0). The amplified phage product was stored at 4 °C.

LB broth medium with *E. coli* ER2738 was dispensed into a 96 well plate for dilution. Serial dilutions (1:10) of the suspended phage products from each selected colony were inoculated on pre-warmed (37 °C) LB agar plates with 3 μL each of IPTG, X-gal, and tetracycline. Phage titer was calculated according to the plaque count on each plate.

Serial dilutions (1:10) of the suspended phage products from each selected colony were mixed into tubes of soft 7.5 g/l agar maintained at 45 °C. Each culture tube was then vortexed, and the soft agar was poured onto LB agar plates. The plates were cooled for five minutes, inverted, and then incubated overnight at 37 °C. Single plaque phages on the plates were counted on the next day.

### DNA Sequencing of Phages

The DNA sequencing was conducted at Molecular Cloning Laboratories (MCLAB, South San Francisco, CA) using rolling circle amplification followed by chain-termination sequencing. The 96 gIII primer was used for sequencing [29].

### ELISA screening to verify phage affinity

ELISA with an HRP-conjugated α-M13 antibody was conducted to verify *in vitro* interaction between selected M13 phages and both native and denatured RAD51A1. The negative control wells were incubated with BSA. The 96-well ELISA plates were coated with 100 μg/mL of either native or denatured RAD51A1 in 0.2 M Na_2_CO_3_ buffer (pH 9.5) and incubated overnight at 4 °C. All wells were subsequently washed with PBST and blocked with 3% BSA for one hour at room temperature. After four washes with PBST, suspended phage solutions from the selected colonies were added and the plates were incubated for one hour at room temperature to allow binding. Four more PBST washes were used to remove unbound phages. To detect bound phages, plates were incubated for two hours with a 1:5000 dilution of HRP-conjugated α-M13 antibody (GE Healthcare) and washed four times with PBST. Negative control wells were incubated with BSA. The Ultra TMB-Blotting Solution was applied to all wells for color development.

### Dot blotting to verify phage affinity

Affinity of the RAD51A1 protein for the selected peptides was verified by dot blotting on nitrocellulose. A nitrocellulose membrane (Thermo Scientific) was prepared with 2 μl of each suspended phage solution and dried at room temperature for 30 min. The membrane was then incubated in 10 mL of blocking buffer (3% BSA in PBS) for one hour at room temperature, followed by three washes of 15 min each with 15 mL of PBST The next incubation was with a 1:1000 dilution of the α-ZmRAD51 antibody from rabbit in PBS with 3% BSA for one hour with gentle agitation. Three washes of 15 min each with 15 mL of PBST followed. Finally, the membrane was incubated with 1:2000 diluted HRP-conjugated goat α-rabbit antibody, washed three times in PBST as previously described, and incubated with 5 mL TMB-Blotting substrate solution with gentle agitation. When color appeared on the dots, the reaction was stopped by adding water.

### Bioinformatic analysis of phage peptide sequences

The phage peptide sequences were used as BLASTp queries in a search of the maize proteome as well as against the set of non-redundant peptides from all available organisms. Uniprot and tBLASTn were also used to screen the selected peptides against known and predicted maize peptides. The pFam database was used to determine whether any of the selected peptides represented known conserved protein domains [7]. BLASTp and ClustalW were both used to align the resulting peptides to the newly confirmed single maize *BRCA2* gene Zm00001d024953_P024, and to RAD51A1.

### Design, synthesis, and binding affinity of synthesized maize peptides

Initial BLASTp results revealed several maize proteins that closely but not identically aligned with the selected phage sequences. The most closely aligned subject sequences from the potentially interacting proteins were identified. These 32 short (13–20 amino acids) peptides were synthesized by Genscript (Piscataway, NJ, USA). The N-terminal was modified by acetylation and C-terminal was modified by amidation. Genscript guarantees purity of at least 85% for each peptide.

Synthesized peptides were dot blotted with RAD51A1 to determine if these synthesized peptide sequences, thought to be present in proteins actually produced by the maize plant, have *in vitro* binding affinity for maize RAD51A1, using the protocol described above.

## Supplementary Information

**Additional file 1: Figure S1**. ELISA screening of phages with native and denatured RAD51A1 proteins. Wells with an OD450 1.5-fold or more above the BSA control were coded as positive. Phages 4, 5, 6, 7, 14, 15, 16, 19, 20, 21, 22 and 23 were positive against both native and denatured protein. Phages 9, 10, 13, and 17 were positive against denatured protein only. **Figure S2**. Phage dot blotting with native RAD51A1. (1) supernatant (2) pellet (3) flow-through (4) first elution and (5) second elution. Phages 5,8,13,14, and 20 bound to the protein supernatant, protein pellet, flow-through, as well as the first elution. This could be due to RAD51A1 present in these stages of the purification process, or due to the fact that the earlier stages of the purification process would contain *E. coli* host proteins and these phages which live in *E. coli* may interact *with E. coli* proteins. High imidazole concentration may have inhibited localization of the protein on the membrane in the second elution, resulting in false negatives. **Table S1**. Proteins from non-maize species with alignments to selected phage peptides in Table 1. **Figure S3**. Dot blotting of 32 synthesized peptides with RAD51A1 on nitrocellulose. Amino acid sequences of all 32 peptides are listed in Table 3. Some peptides were blotted twice. Peptides 1, 2, 3, 7, 8, 11, 12, 15, 18, 19, 20, 25, 30, and 31 bound to RAD51A1. These 14 peptides are listed in Table 4. Note: Two dot blotting experiments were conducted due to the availability of synthesized peptides, GenScript provided two batches of peptide products

## Data Availability

All data generated or analyzed during this study are included in this article and its supplementary information files.

## Abbreviations

DSB: Double-strand break
HRP: Horseradish peroxidase
PBST: Phosphate-buffered saline with Tween® detergent
