# Supplementary material for: Genome-wide investigation of maize RAD51 binding affinity through phage display
Source: BMC Genomics. 2022 Mar 12;23:199. doi: 10.1186/s12864-022-08419-6 (PMC8917730; doi:10.1186/s12864-022-08419-6)
Supplement: Supplementary file 1 — Additional file 1: Figure S1. ELISA screening of phages with native and denatured RAD51A1 proteins. Wells with an OD450 1.5-fold or more above the BSA control were coded as positive. Phages 4, 5, 6, 7, 14, 15, 16, 19, 20, 21, 22 and 23 were positive against both native and denatured protein. Phages 9, 10, 13, and 17 were positive against denatured protein only. Figure S2. Phage dot blotting with native RAD51A1. (1) supernatant (2) pellet (3) flow-through (4) first elution and (5) second elution. Phages 5,8,13,14, and 20 bound to the protein supernatant, protein pellet, flow-through, as well as the first elution. This could be due to RAD51A1 present in these stages of the purification process, or due to the fact that the earlier stages of the purification process would contain E. coli host proteins and these phages which live in E. coli may interact with E. coli proteins. High imidazole concentration may have inhibited localization of the protein on the membrane in the second elution, resulting in false negatives. Table S1. Proteins from non-maize species with alignments to selected phage peptides in Table 1. Figure S3. Dot blotting of 32 synthesized peptides with RAD51A1 on nitrocellulose. Amino acid sequences of all 32 peptides are listed in Table 3. Some peptides were blotted twice. Peptides 1, 2, 3, 7, 8, 11, 12, 15, 18, 19, 20, 25, 30, and 31 bound to RAD51A1. These 14 peptides are listed in Table 4. Note: Two dot blotting experiments were conducted due to the availability of synthesized peptides, GenScript provided two batches of peptide products [file 12864_2022_8419_MOESM1_ESM.docx]

# **Supplementary Material**

## **Figure S1. ELISA screening of phages with native and denatured RAD51A1 proteins.**

Wells with an OD450 1.5-fold or more above the BSA control were coded as positive. Phages 4, 5, 6, 7, 14, 15, 16, 19, 20, 21, 22 and 23 were positive against both native and denatured protein. Phages 9, 10, 13, and 17 were positive against denatured protein only.

##

## **Figure S2. Phage dot blotting with native RAD51A1.**

(1) supernatant (2) pellet (3) flow-through (4) first elution and (5) second elution. Phages 5,8,13,14, and 20 bound to the protein supernatant, protein pellet, flow-through, as well as the first elution. This could be due to RAD51A1 present in these stages of the purification process, or due to the fact that the earlier stages of the purification process would contain *E. coli* host proteins and these phages which live in *E. coli* may interact *with E. coli* proteins. High imidazole concentration may have inhibited localization of the protein on the membrane in the second elution, resulting in false negatives.

## **Table S1. Proteins from non-maize species with alignments to selected phage peptides in Table 1**

| **Phage peptide #** | **Aligned protein annotation** | **E-value** | **Query cover** | **Percent identity** | **NCBI accession** |
| --- | --- | --- | --- | --- | --- |
| 1 | TonB-dependent receptor [*Acinetobacter baumannii*] | 7.4 | 76% | 89% | [WP_000045372.1](https://www.ncbi.nlm.nih.gov/protein/WP_000045372.1?report=genbank&log$=protalign&blast_rank=3&RID=MK69ECW401R) |
| 3 | hypothetical protein AZI85_05800 [*Bdellovibrio bacteriovorus*] | 1.1 | 75% | 83% | [KYG61739.1](https://www.ncbi.nlm.nih.gov/protein/KYG61739.1?report=genbank&log$=protalign&blast_rank=1&RID=MK6AKJTE01R) |
| 4 | PREDICTED: leucine-rich repeat-containing protein 19 isoform X3 [*Poecilia formosa*] | 2.2 | 81% | 55% | [XP_016531137.1](https://www.ncbi.nlm.nih.gov/protein/XP_016531137.1?report=genbank&log$=protalign&blast_rank=1&RID=MK6B6FVU01R) |
| 8 | FAD-dependent oxidoreductase [*Amycolatopsis rubida*] | 4.4 | 72% | 53% | [WP_093572896.1](https://www.ncbi.nlm.nih.gov/protein/WP_093572896.1?report=genbank&log$=protalign&blast_rank=2&RID=MK6E2MJS014) |
| 9 | enterochelin esterase [*Marinomonas mediterranea*] | 2.2 | 81% | 44% | [WP_013660532.1](https://www.ncbi.nlm.nih.gov/protein/WP_013660532.1?report=genbank&log$=protalign&blast_rank=1&RID=MK6EY42Y014) |
| 10 | ABC transporter, integral membrane type 1 [*Penicillium occitanis*] | 1.6 | 93% | 65% | [PCH08546.1](https://www.ncbi.nlm.nih.gov/protein/PCH08546.1?report=genbank&log$=protalign&blast_rank=5&RID=MK6FJJ24015) |
| 11 | structural protein [*Escherichia virus M13*] | 3e-07 | 100% | 100% | [NP_510890.1](https://www.ncbi.nlm.nih.gov/protein/NP_510890.1?report=genbank&log$=protalign&blast_rank=1&RID=MK6G5V3S014) |

A cutoff E-value of 8 was used. The strongest alignment is a perfect match between phage peptide #11 and a structural protein of *Escherichia* virus M13, a suspected false positive.


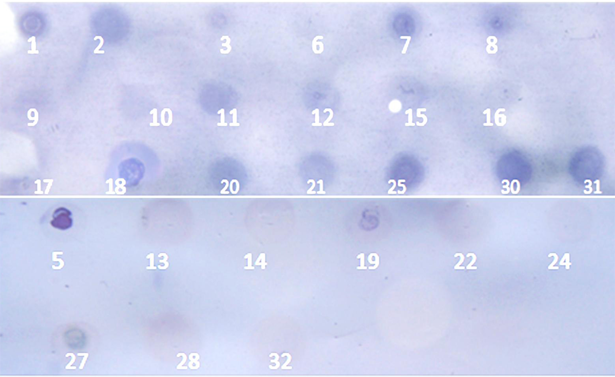


## **Figure S3. Dot blotting of 32 synthesized peptides with RAD51A1 on nitrocellulose.**

Amino acid sequences of all 32 peptides are listed in Table 3. Some peptides were blotted twice. Peptides 1, 2, 3, 7, 8, 11, 12, 15, 18, 19, 20, 25, 30, and 31 bound to RAD51A1. These 14 peptides are listed in Table 4**.** Note: Two dot blotting experiments were conducted due to the availability of synthesized peptides, GenScript provided two batches of peptide products.
